# A simplified evaluation system of surface-related lung lesions of pigs for official meat inspection under industrial slaughter conditions in Germany

**DOI:** 10.1186/1746-6148-10-98

**Published:** 2014-04-27

**Authors:** Thorsten Steinmann, Thomas Blaha, Diana Meemken

**Affiliations:** 1 Field Station for Epidemiology, University of Veterinary Medicine Hannover, Foundation, Buescheler Str. 9, D-49456 Bakum, Germany; 2 Institute for Food Quality and Food Safety, University of Veterinary Medicine Hannover, Foundation, Bischofsholer Damm 15, D-30173 Hannover, Germany

**Keywords:** Risk-based meat inspection, Pneumonia, Reliability, Standardization, Training program, Slaughterhouse, Herd health, Swine, Porcine, Quality assurance

## Abstract

**Background:**

European and national administrative legislation require objective evaluation systems for organ lesions at pig slaughter. These results can be used as basis for herd health improvement programs by farmers and their consulting veterinarians. Various studies have shown that the current evaluation and recording of lesions by authorized meat inspectors are not reliable and produce significant inter-rater disagreement especially for lung lesions in pigs. The objectives of this study were to increase the usability of official meat inspection data by a developed and validated scheme and to analyze potential improvements in the reliability of the proposed system under industrialized slaughter conditions.

**Results:**

A simplified evaluation scheme for surface-related lung lesions was developed based on morphometric evaluations of unaffected lungs with quantitative relationships of each lobe to the whole lung (“Rule of Tens”). Furthermore, a theoretical as well as a hands-on training program for meat inspectors was developed and applied. Based on 5,183 lungs, the authors established a baseline of the inter-rater reliability of current routine assessments of lung lesions as documented by meat inspectors compared with the assessments of an independent veterinarian using the developed simplified evaluation scheme. Most frequent inter-rater disagreements greater than 75% were found for moderate pneumonia. Sources of the deviations most frequently included misinterpretations of technical artifacts, which were erroneously assessed by the meat inspectors as pneumonic lung lesions. Results of the post-training investigation based on 4,646 lungs showed a significantly improved reliability of lung lesion evaluation and the inter-rater agreement increased in all respects. Especially the disagreement of recording moderate cases of pneumonia decreased in total to 15% deviations from reference.

**Conclusions:**

The presented simplified lung evaluation scheme showed its capability to standardize the evaluation of lung lesions according to administrative legislation under industrialial slaughter conditions. The reliability of official meat inspections can be significantly increased with the help of the presented scheme to assess surface-related lung lesions of slaughter pigs. Continuous standardization and optimization can be achieved by personalized training programs in the framework of quality assurance systems for meat inspectors.

## Background

Official controls on products of animal origin intended for human consumption are important for protecting public health as well as animal health and animal welfare. The European Commission regulation calls for continuous improvement of all mentioned aspects, which have to be based on the most recent and relevant information available. This information should constantly adjust current meat inspection practice [1]. Slaughter check results, which are obtained regularly during the slaughter process by official meat inspectors, have increasingly gained significance not only to protect the consumer but also to assess herd health of livestock [2-5]. After implementation of the risk-based meat inspection in the European Union, slaughter check results became important for farmers and veterinarians for several reasons. Firstly, feedback of slaughter check results to the farmers became mandatory in Europe [6]. Secondly, results impact the intensity of the meat inspection method [7-9]. Thirdly, slaughter check results are increasingly accepted as valuable indicators of herd health by farmers and their veterinarians [10-12,4]. Therefore, we report on a quantitative study to increase the reliability of official meat inspection based on a validated, simplified and standardized scheme to assess surface-related lung lesions of slaughter pigs.

According to a national administrative regulation in Germany, an objective evaluation system for organ lesions has to be used for official meat inspections in abattoirs [13]. Outside Germany, diverse evaluation systems for organ lesions are in use. Particularly for lung lesions, various methods and standards are currently in place [14,2,19]. Godwin et al. [14] developed a 55-point lung lesion scheme, which is especially suitable for quantifying lesions caused by enzootic pneumonia in the cranial and medial lobes as well as cranial parts of the caudal lobes. The lung lesion scheme of Madec and Kobisch [15] divides each lobe into quarters, and scores each affected quarter with one point. The minimum score is zero point, i.e. the lung is completely unaltered, and the maximum is 28 points (four points per lobe), i.e. all lobes are completely altered. Straw et al. [17] proposed a simple evaluation scheme for surface areas according to a “Rule of Ten”. However, underlying data in support of this scheme have not been published. Another more sophisticated data-based scheme was published by Christensen et al. [18]. Their scheme evaluates lung lesions depending on the weight of the altered lung lobe and reports the result on a 100-points scale. Any alteration of pars cranialis of lobus cranialis pulmonis sinistri is denoted with five points, of pars caudalis of lobus cranialis pulmonis sinistri with six points, and of the lobus caudalis pulmonis sinistri with 29 points. In the right lung, the lobus cranialis scores eleven points, the lobus medius ten, the lobus caudalis 34, and the lobus accessorius five points, respectively.

The score developed by Blaha [2] assesses pathological-anatomical lung lesions according to the estimating lung alteration. It scores alterations due to pneumonia from low (surface-related extent of lesion ≤ 10%) over moderate (surface-related extent of lesion 11-30%) to high (surface-related extent of lesion > 30%), denoted as Pneumonia 1 to 3 (Pn1, Pn2, Pn3). The German AVVLmH (2009) [12] proposes a modified evaluation scheme adopting the defined grades for inflammatory lung lesions from Blaha [2]. In addition, completely healthy lungs are defined as lungs without lesions (without any observable finding, result key o.b.B.) and merged with lungs having lesions extending to less than 10% of the total surface area (result key PN1) in result category 0. Lungs with lesions from 10 to 30% (result key PN2) are classified in category 1 and lungs with lesions with a surface extent more than 30% (result key PN3) in category 2. Although the German scoring systems are, at first glance, short and easy to handle, various studies have shown that the current evaluation of lung lesions in identical organs by official meat inspectors (specialized authorized veterinarians and qualified assistants) is not reliable, produces significant inter-rater disagreement and unexplainable variation between different abattoirs [4,5]. Variance partitioning coefficients of a recent logistic multilevel analysis with cross-classified random effects of 20 post-mortem findings of official meat inspectors at an Austrian slaughterhouse suggest that especially meat inspection of scalding water lungs can be deemed as not sufficiently standardized [20]. The objective of this study was to increase the reliability of official meat inspection data on the basis of a validated, simplified and standardized scheme to evaluate surface-related lung lesions of slaughter pigs and to analyze potential improvements in the reliability of the proposed under field conditions.

## Methods

### Morphometric quantification and definition of a simplified surface-related evaluation system for lung lesions

**Figure 1:**
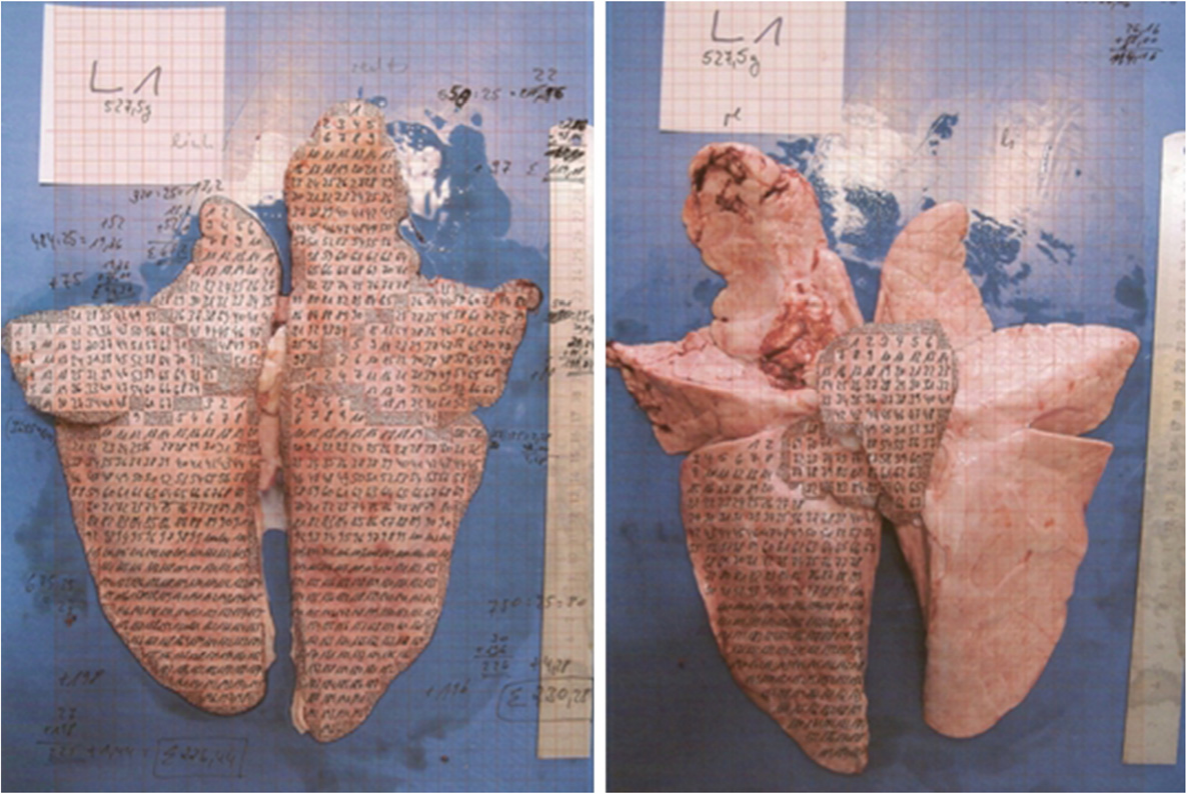
Lung surface morphometry, dorsal and ventral view on cross-section paper.

**Figure 2:**
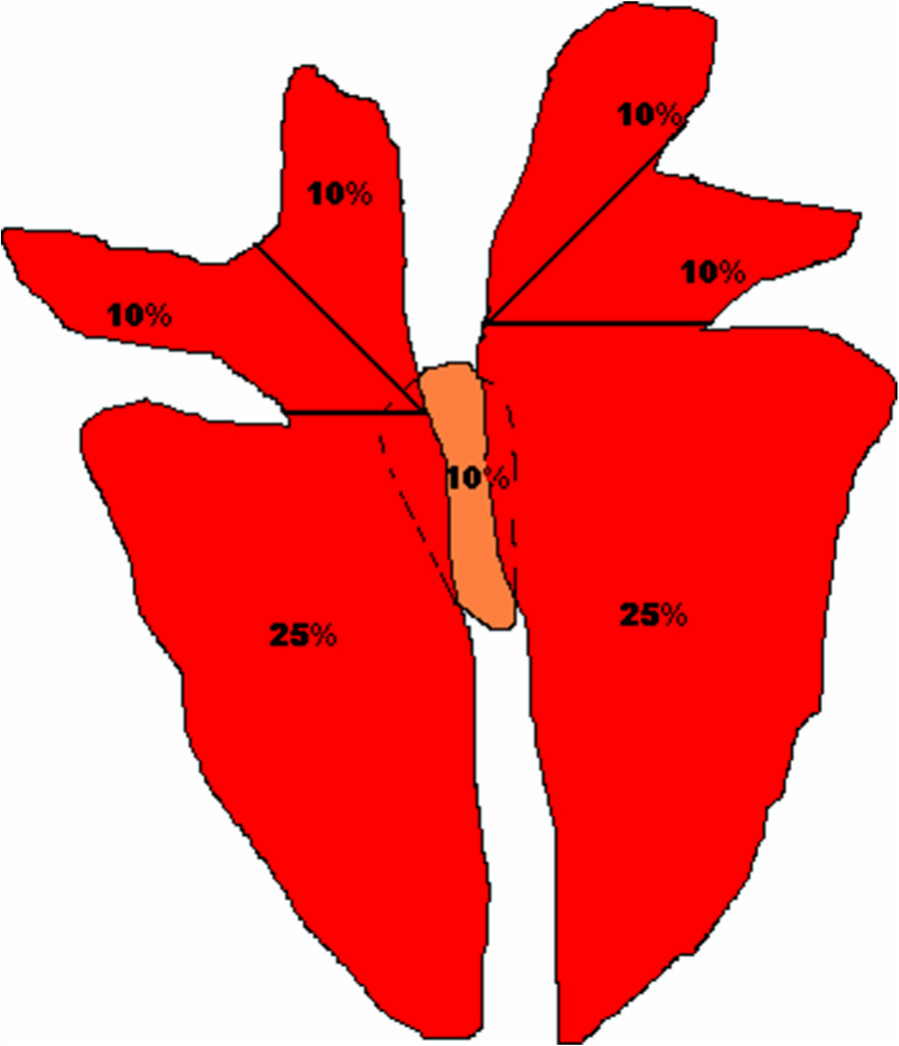
Simplified lung scheme (dorsal) as standardized evaluating base for lung lesions (“Rule of Tens”).

For this purpose, the surfaces of macroscopically unaltered lungs of pigs at slaughter were measured and the quantitative proportion of each lobe to the whole lung was calculated as a percentage of the total lung surface. In March 2011, five unaltered lungs, i.e. all over aerated lungs with a typical light salmon color (Figure 1), from pigs with an average of 100 to 110 kg slaughter weight were selected on a sample basis from a slaughterhouse in Lower Saxony, Germany. Both sides of the lungs were photographed with a digital camera. The distance between the working top and the tripod-fixed camera was 40 cm. The photographs of each lung were printed on cross-section paper (Max Bringmann, Wendelstein, Germany) with the smallest areal sensitivity of 1 mm^2^. Due to the anatomical conditions of the cranial lobes of the lung on both sides, lobes were subdivided along a simulated line with 45° angle based on a horizontal line drawn at the bifurcatio trachealis (Figure 2). Subsequently, the lungs were schematically divided into lung lobes according to their anatomical nomenclature in lobus cranialis pulmonis sinistri pars cranialis and pars caudalis, lobus caudalis pulmonis sinistri, lobus cranialis pulmonis dextri, lobus medius pulmonis dextri, lobus caudalis pulmonalis dextri and lobus accessorius pulmonis dextri [21], respectively. Each of the seven lobes was consecutively numbered on the ventral and dorsal side, and the surface proportion in relation to the total lung surface was calculated. After analyzing the results of the surface calculations of the five unaltered lungs, medians and arithmetic means per lung lobe were calculated. Based on the calculated means, simplifications of the proportion of each lobe were defined as a simplified surface-related evaluation scheme for lung lesions in order to improve operability of the assessments.

### Status-quo investigation

In order to establish a baseline of the inter-rater reliability of current routine assessments, results of routine assessments of lung lesions documented by Authorized Qualified Assistants (AQAs) were compared with the assessment results of an independent observer using the surface-related evaluation scheme. Between July and August 2011, the status-quo investigation of the actual evaluation of lung lesion according to the regulation of the German AVVLmH was performed in an abattoir in North Rhine Westphalia, Germany. The abattoir processed 550 pigs per hour. Pigs were stunned by an automatic electro perfusion, and scalding was performed in a scalding tank. The official slaughter check included scoring of the lung lesions according to the system of the German AVVLmH (2009) [13] and was performed by 15 AQAs, who assessed one lung per five seconds. AQAs were professional butchers or farmers trained according to EC Regulation No 854/2004 [22] which is a non-academic training in Germany. All AQAs were subsequently certified and authorized for meat inspection by the State Government of North Rhine Westphalia, Germany after additional meat inspection seminars of up to 6 months’ duration. AQAs rotated between different positions and workstations every 20 minutes. Lung findings were entered via the touch screen at the slaughter line immediately after the macroscopical examination of each lung. The result keys PN1, 2, or 3 could be selected on the touch screen. As allowed by the German AVVLmH [13], Pneumonia 0 was not scored by AQAs. For this investigation, PN 0 was subsequently calculated (total of examined lungs minus lungs with findings in result keys 1–3). The Observing Veterinarian (OV = T. Steinmann) examined the lungs immediately after the AQAs by using the simplified lung lesion evaluation scheme in combination with the AVVLmH scoring system [13] including a score for PN0. The OV documented the result key manually for predefined time periods in order to synchronize assessment series identical with series assessed by AQAs. The OV remained blinded regarding the entered results of the assistants throughout the period of data collection. During the investigation period of eight slaughter days, lungs of 5,183 randomly selected pigs were scored by AQAs and by the independent OV in parallel. For the comparison, the daily and total sums per result key were used and the deviations were calculated.

### Development and conduct of a specific training program for AQAs

In order to disclose the sources of the calculated inter-rater deviations within the status-quo inquiry, personal discussions between OV and AQAs specific evaluations were performed at the slaughter line. The most frequently analyzed sources of deviations were included in a specific training program for AQAs. The training was divided into two parts: (a) a theoretical training in two sessions lasting four hours each and (b) a practical on-site training of individual AQAs during operations at the slaughter line. The training took place during two weeks in August and September 2012. The core element of the training was the simplified surface-related lung lesion evaluation scheme as an easy-to-use and quick method to quantitatively evaluate pathologically-anatomically lung lesions. This was presented to the AQAs and discussed in detail. Each AQA received a laminated sheet with the scheme to be used at the slaughter line for the training period.

### Investigation of improvements in reliability of evaluation of lung lesions

In this second investigation, the results of post-training assessments of lung lesions by AQAs were compared with the assessment results of the OV, both using the simplified lung lesion evaluation scheme. During four slaughtering days in September 2012, lungs of 4,646 randomly selected pigs scored parallel and independently by 15 AQAs and by the OV as described for the status-quo investigation (see above). The results were compared and the inter-rater reliability was determined based on absolute and relative agreement per result key between AQAs and OV.

## Results

### Morphometric evaluation of ventral and dorsal lung surface areas

Figure 1 shows the morphometric measurement method applied for lung no. 1 (L1) to determine the surface of this lung per lobe and on both sides.

A red square =1 mm^2^.

### Morphometric results of a sample of normal healthy lungs

In Table 1, the morphometric data of the surfaces of each lobe were determined for the ventral and dorsal lobe and both surfaces were combined by cumulation. From five exemplary unaffected lungs, their medians and arithmetic means of the whole surfaces as well as the defined simplified surface proportion as basis for the development of a simplified lung lesion evaluation system for lung lesions are shown.

**Table 1:** Total and relative surface areas of porcine pulmonary lobes

**Lung sample**	**Total surface area**	**Lobus cranialis pulmonis sinistri, pars cranialis**	**Lobus. cranialis pulmonis sinistri, pars caudalis**	**Lobus caudalis pulmonis sinistri**	**Lobus caudalis pulmonis dextri**	**Lobus medius pulmonis dextri**	**Lobus cranialis pulmonis dextri**	**Lobus accessorius pulmonis dextri**
	**cm**^**2**^	**cm**^**2**^**(%)**	**cm**^**2**^**(%)**	**cm**^**2**^**(%)**	**cm**^**2**^**(%)**	**cm**^**2**^**(%)**	**cm**^**2**^**(%)**	**cm**^**2**^**(%)**
L1	462.59	31.70 (6.85)	44.72 (9.67)	111.70 (24.15)	111.32 (24.06)	41.76 (9.03)	60.94 (13.17)	60.45 (13.07)
L2	412.93	39.34 (9.53)	45.57 (11.04)	105.99 (25.67)	86.26 (20.89)	39.34 (9.53)	59.93 (14.51)	36.48 (8.83)
L3	349.10	37.90 (10.86)	30.67 (8.79)	98.72 (28.27)	96.61 (27.67)	38.47 (11.02)	46.77 (13.40)	37.49 (10.74)
L4	538.55	56.95 (10.57)	67.04 (12.45)	146.48 (27.20)	105.25 (19.54)	50.17 (9.32)	56.32 (10.46)	56.34 (10.46)
L5	625.89	46.43 (7.42)	43.49 (6.95)	139.40 (22.27)	168.34 (26.90)	63.00 (10.07)	74.23 (11.86)	91.00 (14.54)
Arithmetic mean of relative surface (%)		9.05	9.78	25.51	23.81	9.79	12.68	11.53
Standard Deviation of Arithmetic mean of relative surface (%)		1.63	1.88	2.14	3.2	0.7	1.39	2.02
Median of relative surface (%)		9.53	9.67	25.67	24.06	9.53	13.17	10.74
Simplified surface proportions (%)		10	10	25 + 25 = 50		10	10	10
Rounding error to median/mean (%)		+0.47 + 0.95	+0.33 + 0.22	−0.46 − 1.02	+0.94 + 1.19	+0.47 + 0.21	−3.17 − 2.68	−0.74 − 1.53

Medians and arithmetic means of sample lobes differed not more than 0.79% per site. The greatest difference of 0.79% was observed for the accessory lobe (cf. Table 1). By using medians, rounding up or down to 10% or 50% for the small lobes or both caudal lobes, respectively, resulted in rounding differences below 0.75% (both caudal lobes taken together). Only the right cranial lobe showed a rounding difference of −3.17% (cf. Table 1).

Figure 2 shows the resulting simplified lung scheme dorsal view with the defined surface proportions of all lung lobes to be used as a standardized method applied to quickly assess how much of a lung surface area is affected by pathological alterations. By summarizing both caudal lobes of the lung (2×25 = 5×10), the simplification follows a “Rule of Tens” (5 × 10 + 10 + 10 + 10 + 10 + 10).

### Status-quo investigation

The absolute and relative differences in total and the deviations from the reference in total, which were collected in two weeks (cf. Table 2), as well as in predefined daily assessment periods (cf. Table 3) were observed. As shown in Tables 2 and 3, the scoring of lung lesions according to the AVVLmH criteria [13] by AQAs differed significantly compared with the assessments of the OV. In the total status-quo observation, the largest overall difference of 5.5% was observed for key PN2 resulting in an over-scoring of + 75.3% deviation from the observer reference. Key PN1 was associated with an under-scoring of - 20.4% in total, whereas key PN3 was associated with an under-scoring of - 11.7% (cf. Table 2).

**Table 2:** Status-quo investigation: evaluations of N = 5183 lungs, total results

**Result key**	**Authorized qualified assistants n (%)**	**Observing veterinarian (reference) n (%)**	**Difference n (%)**	**Deviation from observer (reference = 100) (%)**
PN0	3380 (65.2)*	3402 (65.6)	- 22 (0.4)	- 0.6
PN1	917 (17.7)	1152 (22.2)	- 235 (2.5)	- 20.4
PN2	666 (12.9)	380 (7.3)	+ 286 (5.5)	+ 75.3
PN3	220 (4.2)	249 (4.8)	- 29 (0.6)	- 11.7

*Calculated value, cf. Methods.

**Table 3:** Status-quo investigation: evaluations of N = 5183 lungs, deviation from the observer (reference) per day

	**Status-quo 2011 - deviation from observing veterinarian (reference) per predefined daily period, n (%)**							
Result key	Monday 07.25.	Tuesday 07.26.	Wednesday 07.27.	Thursday 07.28.	Monday 08.22.	Tuesday 08.23.	Wednesday 08.24.	Thursday 08.25.
PN0	−197	−19	−29	18	49	35	87	16
	(−43.8)	(−4.5)	(−6.3)	(5.0)	(15.0)	(7.3)	(20.4)	(3.4)
PN1	66	−53	−2	−4	−48	37	−100	−58
	(43.4)	(−31.9)	(−1.7)	(−3.7)	(−31.6)	(28.0)	(−58.5)	(−37.4)
PN2	122	49	40	18	12	4	4	37
	(348.6)	(87.5)	(121.2)	(30.0)	(20.0)	(7.3)	(8.7)	(105.7)
PN3	9	23	−9	−33	−13	−2	−9	−5
	(32.1)	(63.9)	(−25)	(−47.8)	(−40.6)	(−18.2)	(−33.3)	(−50.0)

*Calculated value, cf. Methods.

**Figure 3:**
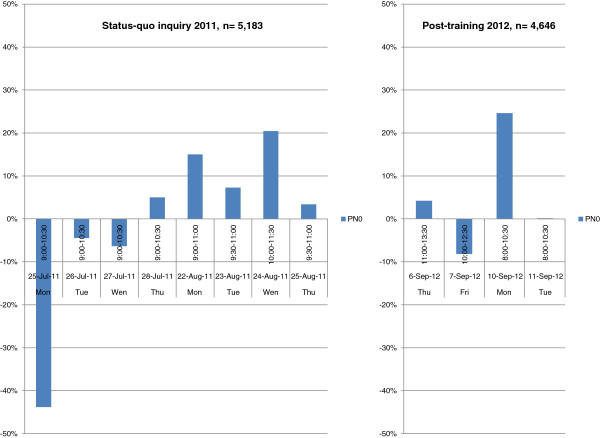
Status-quo and Post-training deviations of AQAs* from the OV (Reference) per day in PN0.

**Figure 4:**
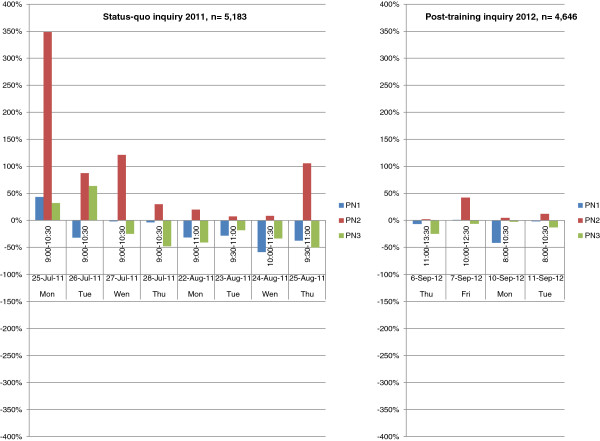
Status-quo and post-training deviations of AQAs to OV (Reference) per day in PN1-3.

The deviations in the daily assessment periods were much larger as shown below (cf. Table 3, Figures 3 and 4). The extent of deviations was largest on the very first day of observation (Monday, 25.07). Subsequently, deviations ranged from an over-scoring of PN2 by 348.6% on the first observation day of the status-quo investigation to an under-scoring of PN3 by 50.0% on the last observation day. Within the keys, PN2 was consistently over-scored ranging from + 7.3% to + 348.6% on all observation days, whereas PN3 was under-scored during the last 6 observation periods ranging from - 18.2% to - 50.0% (cf. Table 3 and Figure 4). For key PN0, Mondays seemed to be generally more burdened with deviations than other days of the week.

### Sources for Under- or Over-scoring and training program for authorized qualified assistants

In preparation of the specific training program, possible objective and subjective sources of status-quo deviations were identified:

1. The reason for the under-scoring of PN1 by AQAs might not be due to their inability to identify low grade pneumonia but due to a misunderstanding resulting in an underestimation of the importance of data collection even for low grade pneumonia and the impact on herd health.

2. The over-scoring of key PN2 by AQAs seemed to be predominantly caused by a misinterpretation of technical artifacts, which were erroneously assessed as pneumonic lung lesions. Especially the ability to differentiate between extensional areal hemorrhages caused by slaughter technique or by disease was remarkably restricted on the part of the AQAs.

3. The individual sensitivity of judging lesions and the power of judgment, which was exhibited by AQAs during the status-quo investigation, was essentially heterogeneous and may depend on psychological and socio-economic factors, including a Hawthorne effect, motivation to work (Monday Blues), cultural differences and lack of understanding to fulfill an essential function in public health and animal welfare.

The training program consisted of a theoretical and a practical part. The theoretical part of the training focused on the components:

**Figure 5:**
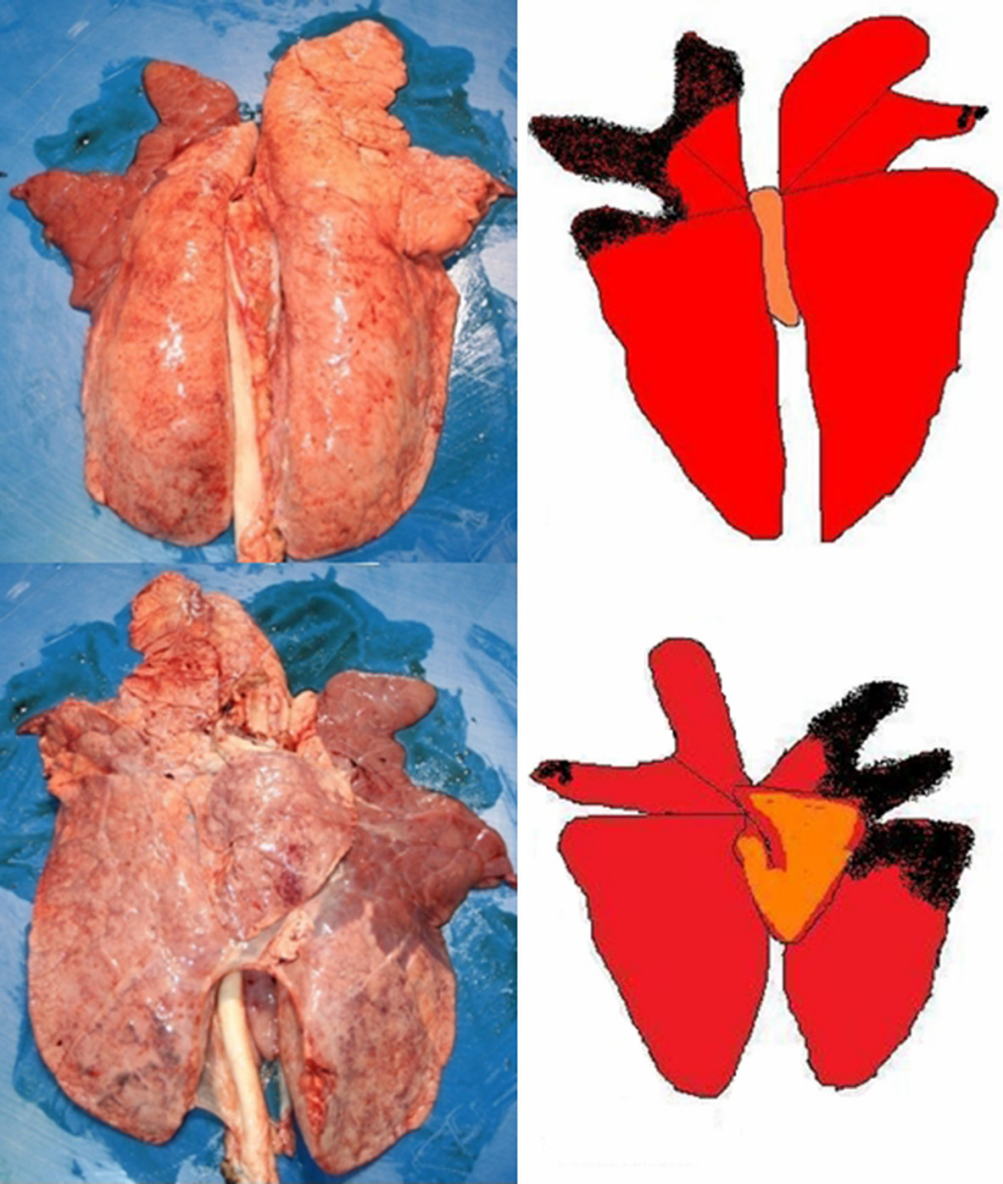
Example of lung photographs and corresponding lung lesions abstracted by colorized schemes (dorsal and ventral).

1. Describing the importance for standardized lung lesion evaluation by showing the aims of informing farmers of the frequencies of lung lesions and the positive effects on herd health caused by a standardized scoring system.

2. Illustrating the anatomical, physiological, histological, immunological and pathological basis of the porcine lung as well as the differentiation of lung alterations caused by slaughter techniques and by diseases.

3. Revising the legal requirements of lung evaluation [7,13].

4. Presenting the developed simplified surface-related lung lesion evaluation scheme to evaluate lung lesions according to its surface as a tool for homogeneous evaluations.

5. Showing affected lungs via photographs and abstracting the relevant lesions by means of a scheme with colorization of the affected lung areas to describe the extent (cf. Figure 5).

The practical part included an individual one-to-one training of each AQA assistant conducted by the OV for 20 hours during the slaughter process at the line. The differences between technical artifacts and pathological lesions were demonstrated by inspection and in doubtful cases by additional palpation and incision of the lung.

### Post-training investigation

The absolute and relative differences in total and the deviations from the reference in total, which were collected in two weeks (cf. Table 2), as well as predefined daily assessment periods (cf. Table 3) were observed. As shown in Tables 4 and 5, the scoring of lung lesions according to the AVVLmH criteria [13] by AQAs improved dramatically and differed at a distinctly smaller degree compared with the assessments of the OV. In the entire post-training observation, an overall difference of 1.5% was observed for key PN2 resulting in an over-scoring of + 15.4% deviation from the observer reference. Key PN1 was associated with an under-scoring of – 12.9% in total, whereas key PN3 was associated with an under-scoring of - 11.5% (cf. Table 4).

**Table 4:** Post-training investigation: evaluations of N = 4646 lungs, total results

**Result key**	**Authorized qualified assistants**	**Observing veterinarian (reference)**	**Difference**	**Deviation from observer (reference = 100)**
	**n (%)**	**n (%)**	**n (%)**	**(%)**
PN0*	2705 (58.2)	2568 (55,3)	+ 137 (3.0)	+ 5.3
PN1	1254 (27.0)	1440 (31.0)	- 186 (4.0)	- 12.9
PN2	526 (11.3)	456 (9.8)	+ 70 (1.5)	+ 15.4
PN3	161 (3.5)	182 (3.9)	- 21 (0.5)	- 11.5

*Calculated value, cf. Methods.

**Table 5:** Post-training investigation: evaluations of N = 4646 lungs, deviation from the observer (reference) per day

	**Post-training 2012 - deviation from observing veterinarian (reference) per predefined daily periods, n (%)**			
Result key	Thursday	Friday	Monday	Tuesday
	06.09.	07.09.	10.09.	11.09.
PN0	29 (4.2)	−48 (−8.1)	157 (24.5)	−1 (−0.2)
PN1	−21 (−6.7)	4 (1.0)	−162 (−41.5)	−7 (−2.0)
PN2	2 (2.1)	47 (42.3)	6 (4.7)	15 (12.4)
PN3	−10 (−25.0)	−3 (−6.1)	−1 (−2.6)	−7 (−13)

The deviations within the daily assessment periods decreased as shown below (cf. Table 5, Figures 3 and 4). The extent of deviations did not exceed 50% and were largest on weekend-related days (Friday, 07.09 and Monday, 10.09.). Deviations ranged from an over-scoring of PN2 by + 42.3% to an under-scoring of PN1 by - 41.5%. Among the keys, PN2 was again consistently over-scored ranging from + 2.1% to + 42.3% on all observation days, whereas PN3 was slightly, but consistently under-scored during the whole post-training observation periods ranging from – 2.6% to - 25.0% (cf. Table 5 and Figure 4). For key PN0, a Monday seemed to be generally more burdened with deviations (over-scoring by + 24.5%) than other days of the week.

## Discussion

The authors developed and validated a simplified and standardized scheme in order to assess surface-related lung lesions of slaughter pigs according to the German AVVLmH administrative regulation.

Morphometric data of a sample of five lungs showed that medians and arithmetic means did not differ significantly from each other suggesting that their surfaces were nearly symmetrically distributed and that both, mean and median could be used for quantitative simplifications. In order to achieve an easy-to-use numerical aggregation rule for evaluating lung lesions the “Rule of Tens” was developed. Estimation rules are regarded as highly effective for approximations under time pressure, e.g. in medical emergencies such as burns. A “Rule of Nines” is used in human medicine to determine severely burned body surface areas in adults using multiples of 9 and to guide treatment decisions [23]. Already Straw et al. [17] proposed breaking the small porcine lung lobes down to 10%. However, morphometric data in support of this approximation and the method how to estimate surfaces (from ventral, dorsal or both) could not be found in the literature. Here, the authors provide data supporting this concept and extend it to an overall “Rule of Tens” for pig lungs. The advantage of this standardized estimation method for lung surfaces is that it is rapidly realizable and feasible to apply without expensive or sophisticated technical tools or instruments. Furthermore, due to the determination that the cranial lobes are separated from each other by an imaginary line with an approximate 45° angle, this method was proven to have high practicability and usefulness during this study.

A limitation of the method might be that the used two-dimensional photograph does not represent the three-dimensional lung tissue precisely. Nonetheless, according to Hill et al. [24] and Davies et al. [25], who quantified differences between two-dimensional and three-dimensional measuring methods of lungs, the agreement is remarkably high. In addition, the lack of precision is negligible due to the purpose to develop a standard scheme for measuring lung lesions in industrial abattoirs. Godwin et al. [14] developed a lung lesion evaluation scheme focused on lesions typically caused by enzootic pneumonia. The main deficits of this evaluation system are that lesions located in the diaphragmatic lobes as well as chronic lesions are not recorded, so that this system may overestimate the respiratory herd health status. The benefit of the scheme by Madec and Kobisch [15] is that for application an extra surface standard is not necessary. This is due to the imaginary subdivision of each lobe into quarters which is adequately accessible by inspection. The limitation of their system is that the relationship between the total number of the score points and the extent of the affected lung surface does not represent the realistic lung proportion, i.e. a totally affected cranial or medial lobe and a totally affected caudal lobe results in equal score points although the proportions are different. Furthermore, the evaluation as well as the documentation of the findings for each of the seven lung lobes are too time-consuming so that the scheme by Madec and Kobisch [15] is more suitable for a laboratory setting.

An advantage of the scheme by Christensen et al. [18] is that it seems to be the most realistic one because they base their proportions on precise volume measurements of the different lung lobes. This leads to a distinctive lung dimorphism between the left and right lung, whereas the right lung is larger than the left lung. Especially the right cranial and medial lung lobes are nearly double as large as the left cranial lobes. The most substantial disadvantage of that scheme is reasoned in its difficult applicability at slaughter line especially under field conditions. In addition, the evaluation of lung lesions in volume is impossible without incising the tissue.

The subsequent status-quo investigation clearly revealed room for improvement. It is well known that participant observation can only do so much for the research as the sole presence of the observer in the field influences the participants' behavior. For this reason, we avoided any intervention or change in the working process of the AQAs as far as it was technically possible. We achieved a type of data collection characterized by a passive participation of only a single Observing Veterinarian in the bystander role. To overcome limitations regarding the ability to establish rapport, a separate and blinded documentation system was implemented for the Observing Veterinarian, and the assessment comparisons were conducted based on predefined observation time-frames with incomplete overlapping of large lung samples rather than predefined small sizes of lungs samples with incomplete overlapping of observation times. The design of our observational methods implied compromises that did not support a rationale for calculating intra-observer repeatability and inter-observer reproducibility based on correlation statistics. Descriptive statistics were regarded as being more appropriate for the approach of this study. By using this means, an impact of the observer involvement in terms of a distinct effect on the result key PN0 and PN2 was seen on day 1 of the status-quo investigation (cf. Figures 3 and 4). This effect bears a resemblance to the Hawthorne effect as a form of reactivity whereby observed persons improve or modify their behavior in response to the fact that they know they are being observed [26]. It may be assumed that participating AQAs acted differently under observation with the aim of achieving a similar result to that of the Observing Veterinarian. This observational artifact obviously led the AQAs to an own interpretation of the purpose of the status-quo investigation and seemed to changed their behavior to fit that interpretation. A possible reason for these characteristics is the expectation of AQAs that they will be evaluated and thus they figured out how to control the observation and to attain good scores. The Hawthorne effect might have been facilitated to appear in the status-quo investigation as the German AVVLmH administrative regulation allows low grade pneumonia to be scored in result category 0 implying an irrelevant finding. This was the routine at the investigated slaughter line, but this routine obviously reduces scoring sensitivity for pathological alterations. It is important to note that the presumed strong Hawthorne effect at the beginning of the status-quo investigation became smaller in the course of the investigation. This effect can be seen in relation to ostensibly objective causes like misinterpretations of technical artifacts, which were erroneously assessed as pneumonic lung lesions, and the limited ability to differentiate between extensional pre- and post-mortal hemorrhages. It is conceivable that latter causes might play a greater role in the course of the status-quo investigation. The fact that these significant disagreements occurred mainly within the critical discrimination of keys PN0 and PN2 is seen as a matter of concern by the authors and, retrospectively, justified a specific and efficient training of the AQAs. Although the described misinterpretations of artifacts by the AQAs were at least subjective appraisals of the Observing Veterinarian, but he as a veterinarian has profound theoretical knowledge and practical skills in veterinary pathology backed up with knowledge about lung lesions and its histopathology. Nonetheless incorrect scoring by the Observing Veterinarian cannot be precluded but the frequency of wrong lung assessments ought to be much lesser compared to AQAs.

The specific training followed established didactical principles and separated a theoretical part from a practical one. A thorough theoretical revision of specific patho-anatomical details of the lung and its lesions as an important prerequisite for evaluating pneumonia was strongly appreciated by the AQAs. The colorized abstractions of pneumonic lesions which were put in context with corresponding photographs (cf. Figure 5) supported the detectability of lesions and their extensions caused by pneumonia. It was confirmed by the AQAs that the simplified lung lesion evaluation scheme strongly improved the evaluation of the affected lung surface in the sense of an easy-to-use tool with the potential to enormously simplify the graduation of lung lesions into low, medium and high even under high time pressure and stress. Questions during the course about differentiation of alterations either caused by disease or by slaughter techniques were discussed directly at the slaughter line with the AQAs on a personal case-by-case basis. In doubtful cases, lungs were palpated and incised as proposed by Nathues et al. [27]. The authors were aware that this positive feedback to the training given by the AQAs could have been partially the result of polite responses. Thus, it became also an objective of the post-training investigation to check this particular question.

In general, the post-training investigational data suggested a significant improvement potential compared with the status-quo investigation. Particularly, the critical keys PN1, 2 and 3 exhibited a major increase in reliability as judged by the AQAs. It seemed that the previously suggested Hawthorne effect, which was apparently associated with keys PN0 and PN2 in the status-quo investigation, had nearly vanished. This change might be one of the specific positive results of the personalized practical training triggered by and due to the sympathy and interest of the Observing Veterinarian. Further discussion of this possible correlation may lead to an improved understanding of a management effect at real-world industrial slaughter lines with the important question how management can make AQAs perform better because they feel better.

Nevertheless, results of the post-training investigation were far from ideal. Deviations from the observer continued to revealed differences of greater than 10% in several periods for the critical keys PN2 and 3, and deviations peaked again slightly on working days close to the weekend. Further investigations of these remaining deficits, possibly based on unannounced spot checks, and data-derived trainings of AQAs seem to be an option to further increase the reliability of the current practice in meat inspections at industrial slaughter lines.

## Conclusion

It may be concluded that the reliability of official meat inspections in a real-world industrialized setting can be increased with the help of i) a validated, simplified and standardized scheme to assess surface-related lung lesions of slaughter pigs (“The Rule of Tens”) and ii) an accompanying theoretical and practical training of authorized qualified assistants (AQA) in evaluating pathological lung lesions. A continuative standardization and optimization can be realized by repetitive and individual training programs based on controls within the framework of quality assurance.

## Abbreviations

AQAs: Authorized qualified assistants = synonymous to official auxiliaries in Reg. (EC) No. 854/2004; OV: Observing veterinarian (Reference).

## Competing interests

The authors declare that they have no competing interests.

## Authors’ contributions

TS participated in the study design and carried out the data collection, developed the simplified standard surface area scheme, conducted the training program and drafted the manuscript. TB and DM planned and designed the study, coordinated the research project and participated in drafting the manuscript. TS, TB and DM performed the statistical analysis and were involved in the interpretation of data. All authors read and approved the final manuscript.
